# Shenmai injection ameliorates doxorubicin-induced myocardial injury by suppressing autophagy-apoptosis via miR-30a

**DOI:** 10.18632/aging.205188

**Published:** 2023-11-07

**Authors:** Yanyang Li, Lu Fan, Xiaoming Wang, Shichao Lv

**Affiliations:** 1 Department of Integrated Traditional and Western Medicine, Tianjin Medical University Cancer Institute and Hospital, Tianjin 300060, China; 2 National Clinical Research Center for Cancer, Tianjin 300060, China; 3 Key Laboratory of Cancer Prevention and Therapy, Tianjin 300060, China; 4 Tianjin’s Clinical Research Center for Cancer, Tianjin 300060, China; 5 First Teaching Hospital of Tianjin University of Traditional Chinese Medicine, Tianjin 300381, China; 6 National Clinical Research Center for Chinese Medicine Acupuncture and Moxibustion, Tianjin 300381, China

**Keywords:** cardiotoxicity, doxorubicin, autophagy, apoptosis, Shenmai injection

## Abstract

Context: Autophagy-apoptosis is the core mechanism of doxorubicin-induced myocardial injury. miR-30a is a pivotal factor in the regulation of autophagy and apoptosis. It remains unclear whether SMI exerts cardioprotective effect by regulating autophagy and apoptosis via miR-30a.

Objective: This study evaluates the effects of SMI on ameliorating doxorubicin-induced myocardial injury.

Materials and Methods: The level of LDH and CK, and the expression of miR-30a was detected. mCherry-EGFP-LC3B double fluorescence was used to observe autophagy flow. Apoptosis was detected by Annexin V/PI staining. Western Blot was used to estimate the expression of autophagy related proteins and apoptosis-related proteins.

Results: Compared with the control group, there were evidently decreased cell viability, elevated level of LDH and CK, down-regulated expression of miR-30a in the model group. Data from Western blot and fluorescence indicated that doxorubicin contributed to the elevated autophagy and apoptosis. Compared with the model group, there were increased cell viability, decreased level of LDH and CK, and up-regulated expression of miR-30a in the Shenmai group and the Shenmai + miR-30a inhibitor group. Meanwhile, the results manifested that there were suppressed autophagy flow accompanied by the down-regulated expression of Beclin-1, LC3-II, LC3-II/LC3-I and up-regulated expression of p62 protein, and declined apoptosis rate accompanied by the up-regulated Bcl2 expression and the down-regulated expression of Bax, Cleaved Caspase-9, Cleaved Caspase-9/Caspase-9, Cleaved Caspase-3, Cleaved Caspase-3/Caspase-3 in the Shenmai group and the Shenmai + miR-30a inhibitor group.

Discussion and Conclusion: Shenmai injection inhibited autophagy and apoptosis via miR-30a, thereby alleviating doxorubicin-induced myocardial injury.

## INTRODUCTION

Cardiotoxicity, the main cause of increased mortality in tumor survivors and the most serious side effect of antineoplastic medications, has passive influence on the effectiveness of antineoplastic drugs [1]. Cardiotoxicity is commonly found in Anthracyclines (Doxorubicin, Epirubicin), Alkylating agents (Cyclophosphamide, Cisplatin), Anti-metabolites (Fluorouracil), Anti-microtubular drugs (Paclitaxel, Vincristine), and targeted drugs (Trastuzumab, Bevacizumab), and among them, anthracyclines are the most common offenders [2]. Anthracyclines are the cornerstone of chemotherapy for their extensive anti-tumor spectrum and potent anti-tumor effects. Nevertheless, cardiotoxicity, the dose-limiting toxicity of anthracyclines, is the most frequent and severe toxicity in clinical application. Studies have proved that cumulative dosage of doxorubicin, the classic anthracycline, raised the risk of cardiotoxicity. Therefore, doxorubicin-induced cardiotoxicity or myocardial injury models in zebrafish, rabbits, and rats came along successively [3–6]. *In vitro*, myocardial injury models are included by hypoxia/reoxygenation, hypertrophy, oxidative stress, inflammation, internal environment disturbance, and toxicity, and among them, doxorubicin is the most frequently used method [7].

Shenmai Injection (SMI), derived from Traditional Chinese Medicine prescription Shengmai Pander, is made of Ginseng Radix et Rhizoma Rubra (Panax ginseng C. A. Mey, Hongshen) and Radix Ophiopogonis (Ophiopogon japonicus (Linn. f.) Ker-Gawl, Maidong) combined with modern production techniques, which is wildly used for the treatment of tumors and cardiovascular diseases. A meta analysis of 14 studies involving 1105 patients showed that the application of Shenmai injection was beneficial in preventing and treating cardiotoxicity caused by chemotherapy for malignant tumors [8]. Another Meta-analysis of 15 articles including 2042 patients indicated that Shenmai injection could reduce the abnormal changes of electrocardiogram, the high expression of myocardial enzymes, the incidence of arrhythmia, and the grading of cardiac adverse reactions [9]. It has been reported that SMI improved cardiac output and ejection fraction of rats with doxorubicin-induced myocardial injury, decreased serum AST, lactate dehydrogenase (LDH), and CK-MB levels, raised the expression of IL-10 and reduced IL-6, TNF-α, and NF-κB protein levels [10]. In chronic heart failure rat models induced by the ligation of left anterior descending coronary artery, SMI seemed reduce serum brain natriuretic peptide (BNP), high-sensitivity C-reactive protein (CRP), and cyclic citrullinated peptide (CCP) levels, as was myocardial fibrosis [11]. In ischemia-reperfusion injury rat models, the application of SMI relieved myocardial apoptosis, boosted the expression of Bcl2, and decreased the expression of Bax and Caspase-3 [12]. In the oxidative damage model of H9c2 cells constructed under the hypoxia conditions of 95% N2 and 5% CO2, SMI increased the cell survival rate and the activity of SOD, and decreased the levels of CAT, MDA and H_2_O_2_ [13]. The apoptosis rate of HUVECs cell injury model induced by lipopolysaccharide was decreased, and the expression of adhesion factors and inflammatory factors, such as TNF-α, IL-6 and IL-1β were inhibited [14]. Above studies indicated that Shenmai Injection reduced myocardial injury, delayed ventricular remodeling, and improved cardiac function. The underlying mechanism is related to the inhibition of inflammation and apoptosis, but the multi-component, multi-pathway, and multi-target properties of SMI still require further clarification. Our earlier research included 19 randomized controlled trials with 2,331 participants to evaluate the efficacy of Shengmai San in the treatment of cardiotoxicity of anthracyclines. The results showed that the treatment group was superior to the control group in improving arrhythmia, reducing LVEDD and LVESD, and decreasing myocardial enzymes such as CK [15]. We demonstrated that SMI boosted the expression of miR-30a, inhibited myocardial autophagy, and ameliorated doxorubicin-induced cardiac damage *in vivo* [16]. Whereas the specific targets and mechanisms of SMI remain unclear. To gain evidence that the protective effects of SMI were mediated by miR-30a, we treated H9c2 cells with doxorubicin combined with the miR-30a inhibitor.

## MATERIALS AND METHODS

### Drugs and reagents

H9c2 cells (Beina Biotechnology Co., Ltd., BNCC337726), Shenmai Injection (Chiatai Qingchunbao Pharmaceutical Co., Ltd., 2104127), Doxorubicin hydrochloride for injection (Shenzhen Main Luck Pharmaceuticals Inc., 1905E1), miR-30a inhibitor (Sangon Biotech (Shanghai) Co., Ltd., R15701), Fetal bovine serum (FBS) (Gibco, 10099-141), DMEM medium (Gibco, C11995500BT), Cell counting kit-8 (CCK-8) (Dojindo, CK04), LDH activity assay kit (Beyotime Biotech Inc., C0016); Creatine kinase (CK) enzyme-linked immunosorbent assay (ELISA) kit (Shanghai Fankewei Co., Ltd., F3651-A), pBABE-puromCherry-EGFP-LC3B (Addgene, 22418), AnnexinV-FITC apoptosis detection kit (Nanjing Keygen Biotech Co., Ltd., KGA108), RIPA lysis buffer (Beijing Solarbio Science and Technology Co., Ltd., R0020), BCA protein assay kit (Beyotime Biotech Inc., P0012), SDS-PAGE gel kit (Beijing Solarbio Science and Technology Co., Ltd., P1200), Prestained color protein ladder (Beijing Solarbio Science and Technology Co., Ltd., P0077), ECL luminescence reagent (Monad Biotech Co., Ltd., PW30701S), Bcl-2 antibody (Beijing Biosynthesis Biotechnology Co., Ltd., s-0032R), Bax antibody (Proteintech Group, 50599-2-Ig), Caspase-3 antibody (Cell Signaling Technology, Inc., 14220T), Caspase-9 antibody (Cell Signaling Technology, Inc., 9508T), Beclin-1 antibody (Cell Signaling Technology, Inc., 3495T), p62 antibody (Abcam Plc, ab109012), LC3 antibody (Cell Signaling Technology, Inc., 12741), β-tubulin antibody (Beijing Zhong Shan -Golden Bridge Biological Technology Co., Ltd., TA-10), Goat anti-mouse IgG-HRP (Beijing Zhong Shan -Golden Bridge Biological Technology Co., Ltd., ZB-5305), Goat anti-rabbit IgG-HRP (Beijing Zhong Shan -Golden Bridge Biological Technology Co., Ltd., ZB-5301), TRNzol Universal (Tiangen Biotech (Beijing) Co., Ltd., DP424), MiRcute plus miRNA qRCR kit (Tiangen Biotech (Beijing) Co., Ltd., FP411), miRcute plus miRNA First-strand cDNA kit (Tiangen Biotech (Beijing) Co., Ltd., KR211), Applied Biosystems™ MicroAm™ Fast Optical 96-Well Reaction Plate with Barcode (ABI, 4346906), Applied Biosystems™ MicroAmp™ Optical 96-Well Reaction Plate with Barcode and Optical Adhesive Films (ABI, 4314320).

### Cell culture and treatments

H9c2 cells were grown in DMEM medium containing 10% FBS with 5% CO_2_ at 37°C. Medium was replaced every 2–3 days, the logarithmic-phase H9c2 cells were divided into the control group (routine culture + inhibitor-miR NC), the model group (doxorubicin + inhibitor-miR NC), the Shenmai group (doxorubicin + SMI + inhibitor-miR NC), the Shenmai + miR-30a inhibitor group (doxorubicin + SMI + miR-30a inhibitor). In addition to the control group, the rest three groups were treated with 1500 nM doxorubicin for 8h, and each group was transfected with Lipofectamine™ 2000. Firstly, 100 pmol/well inhibitor-miR NC and inhibitor-miR-30a were respectively diluted in 50 μL Opti-MEM. 5 μL/well Lipofectamine™ 2000 was diluted in 50 μL Opti-MEM. After the incubation for 5 min, the diluted DNA and diluted Lipofectamine™ 2000 were mixed gently and incubated for 20 min at room temperature. The complexes were added to relevant well and incubated for 16 h (at 37°C and 5% CO_2_). Then the appropriate cells treated with 6.25 μL/mL SMI for 24 h.

### Cell viability

CCK-8 assay was used to detect the viability of cardiomyocytes. The suspension of logarithmic-phase H9c2 cells (100 μL/well) was inoculated in 96-well plate (at 37°C, 5% CO_2_). After treatment, CCK-8 solution (10 μl) was added to each well and incubated at 37°C for 1 h. The optical density (OD) values, detected at 450 nm with a microplate reader, were applied to assess the cell viability.

### LDH activity assay

LDH is a stable glycolytic enzyme in the cytoplasm. When the cell is attacked, the permeability of the cell membrane increases, and LDH is rapidly released outside the cell. The activity of LDH in the cellular supernatant reflects the degree of the change in the permeability of the cell membrane. The H9c2 cells were inoculated in the 24-well plate. After treatment and centrifugation at 500 r/min for 5 min, the supernatant was incubated with 60 μL LDH working reagent for 30 min, and measured according to the manufacturer’s instructions. Results were obtained at 490 nm by a spectrophotometer. LDH leakage (%) = (treated sample absorption- sample control well absorption)/ (absorbance of the maximum enzyme activity of the cell- sample control well absorption) × 100.

### ELISA assay

H9c2 cells were inoculated in the 24-well plate and centrifuged at 1500 r/min for 20 min after treatment. The standard and sample were added to each well and incubated at 37°C for 30 min. Removing the liquid without wash, reagents were added to each well. After laminating, incubating and washing, the TMB substrate was added to each well and incubated for 10 min avoiding light. Then stop solution was added to each well. The OD values detected at 450 nm with a microplate reader. And the CK level was calculated according to the standard curve. Serum myocardial enzyme profile is an important index for the diagnosis of myocardial injury. The increase of CK-MB is the main cause of myocardial cell injury, and the total CK will also increase.

### Quantitative real-time polymerase chain reaction (RT-qPCR)

H9c2 cells were inoculated in a culture dish, the total RNA was extracted by TRIzol and then reversely transcribed into cDNA. QRT-PCR was performed by the SYBR Green method. The reaction condition: predenaturation at 95°C for 15min; denaturation for 20 s at 94°C, anneal and extension at 60°C for 34 s, and 40 cycles were performed. Based on the RT-q PCR results, the relative mRNA level of miR-30a were evaluated by the 2^−ΔΔCt^ method. The primers were designed and synthesized by Jinweizhi Biotechnology Co. Ltd. (Suzhou, China). Primer sequences were as follows: miR-30a: 5′-CGCTGTAAACATCCTCGACTGGAAG-3′. U6: 5′-CTCGCTTCGGCAGCACA-3′ (F) and 5′-AACGCTTCACGAATTTGCGT-3′ (R).

### mCherry-EGFP-LC3B fluorescence

The H9c2 cells were inoculated in the 24-well plate and transfected with mCherry-EGFP-LC3B plasmid (referring to 1.2). After transfection and treatment, cells were washed twice in PBS and the supernatants were discard. The cells were fixed with 40 g/L paraformaldehyde for 30 min and washed three times with PBS. And then the cells were collected and analyzed by laser confocal microscope. In general, mCherry-GFP-LC3B exists in the cytoplasm as diffuse yellow fluorescence that indicates non-autophagy (the fusion of mCherry and GFP); mCherry-GFP-LC3B accumulates on the autophagosome membrane in the form of yellow spots that indicates autophagy; the fusion of autophagosome and lysosome presents as red spots partially because of the quenching of GFP fluorescence.

### Annexin V/propidium iodide (PI) staining

H9c2 cells were inoculated in a culture dish. Cells were washed twice with PBS after treatment (referring to 1.2). Discarding the supernatant, and the sediment of cells was resuspended with 500 μL Binding Buffer. Each well was added to 5 μL Annexin V-FITC and 5 μL PI following the instructions and incubated for 15 min at room temperature. Flow cytometry was used to analyze stained cells. The apoptosis rate (%) = cells of Q2 quadrant (%) + Q4 quadrant cells of Q4 quadrant (%).

### Western blot analysis

H9c2 cells were inoculated in a culture dish. Proteins were extracted using RIPA lysis buffer after treatment, and then centrifuged at 12000 r/min for 20 min to obtain supernatants. The protein concentration was determined by BCA. The proteins were separated on SDS–polyacrylamide gels and electro-transferred to polyvinylidene difluoride membranes. After blocking in 5% skim milk for 1 h, membranes were incubated with primary antibodies: Bcl-2 antibody (dilution 1:1000), Bax antibody (dilution 1:4000), Caspase-3 antibody (dilution 1:1000), Caspase-9 antibody (dilution 1:1000), Beclin-1 antibody (dilution 1:1000), LC3 antibody (dilution 1:1000), p62 antibody (dilution 1:20000), β-tubulin antibody (dilution 1: 2000) were incubated overnight at 4°C. Then the HRP labeled secondary antibody (1:5000) was incubated at room temperature for 1 h. The proteins were observed by ECL luminescence solution and analyzed through Image J software, and the relative expression of the target protein was calculated with β-tubulin as the internal reference. The relative expression of the target protein = (grey level of target protein - grey level of control protein)/(grey level of β-tubulin - grey level of control protein).

### Data analysis

SPSS 17.0 software was used for statistical analysis. The data were described by $\bar{x}\pm \text{s}.$ One-way analysis of variance and LSD test were used for comparison among multiple groups. *P* < 0.05 was considered statistically significant.

## RESULTS

### Appropriate concentration of doxorubicin for inducing H9c2 cells damage

**Figure 1:**
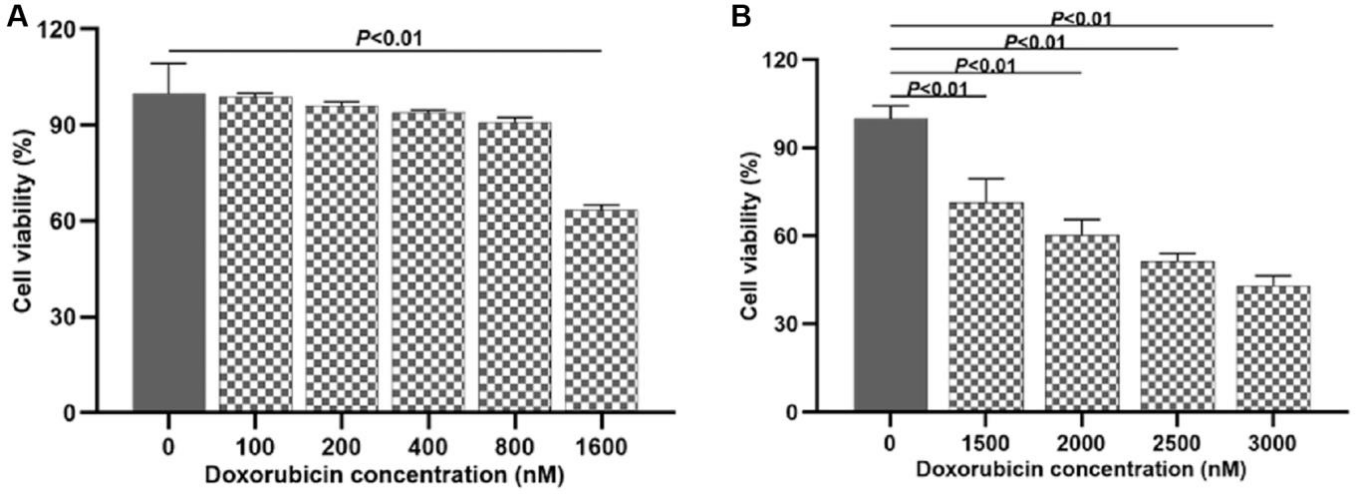
**Investigating the suitable dosage of doxorubicin to induce H9c2 cell injury.** (**A**) Effects of doxorubicin on H9c2 cells viability at different concentrations (0~1600 nM); (**B**) Effects of doxorubicin on H9c2 cells viability at different concentrations (1500~3000 nM).

CCK-8 assay was used to detect the viability of H9c2 cells 24 h after the administration of doxorubicin at different concentrations (0, 100, 200, 400, 800 and 1600 nM). The results turned out that compared with the control group, there was no significant variation in terms of H9c2 cells viability when the concentration of doxorubicin was 0~800 nM (*P* > 0.05); when the concentration of doxorubicin was 1600 nM, the cell viability declined (*P* < 0.01). According to the above results, we further detected the effect of doxorubicin at different concentrations (1500, 2000, 2500 and 3000 nM) on cell viability. The results showed that as the dosage of doxorubicin increased, the cell viability decreased by degrees (*P* < 0.01) in a dose-dependent manner (Figure 1A, 1B). Ultimately, 1500 nM of doxorubicin was optimized for subsequent experiments.

### Effects of Shenmai injection at different concentrations on the viability of normal H9c2 cells

**Figure 2:**
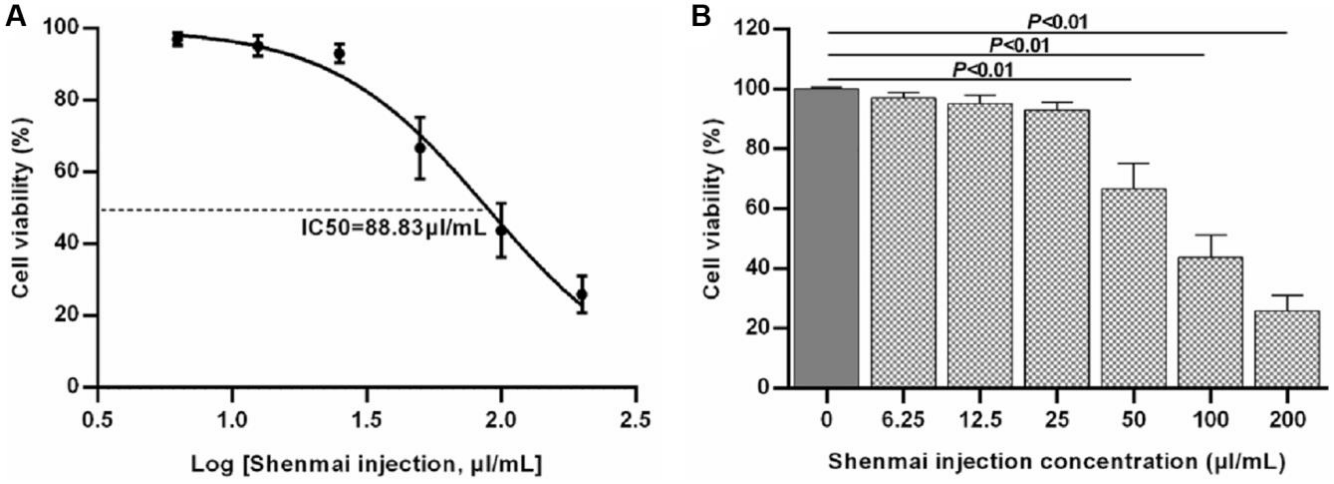
**Effects of Shenmai injection at different concentrations on normal H9c2 cell viability.** (**A**) Fitting curve of cell viability; (**B**) Effects of Shenmai injection on the viability of normal H9c2 cells with different concentrations.

CCK-8 assay was used to detect the effects of Shenmai injection at different concentrations (0, 6.25, 12.5, 25, 50, 100 and 200 μL/mL) on the viability of normal H9c2 cells. The results indicated that compared with the control group, there was no significant variation in terms of H9c2 cells viability when the concentration of Shenmai injection was 0~25 μL/mL (*P* > 0.05). When the concentration of Shenmai injection was 50~200 μL/mL, the cell viability decreased evidently (*P* < 0.01), and with the elevated concentration of Shenmai injection, the cell viability appeared a downward trend (Figure 2A, 2B). Based on the above data, 6.25, 12.5 and 25 μL/mL were chosen as the proper concentration of Shenmai injection for further study.

### Effects of Shenmai injection at different concentrations on the viability of injured H9c2 cells

**Figure 3:**
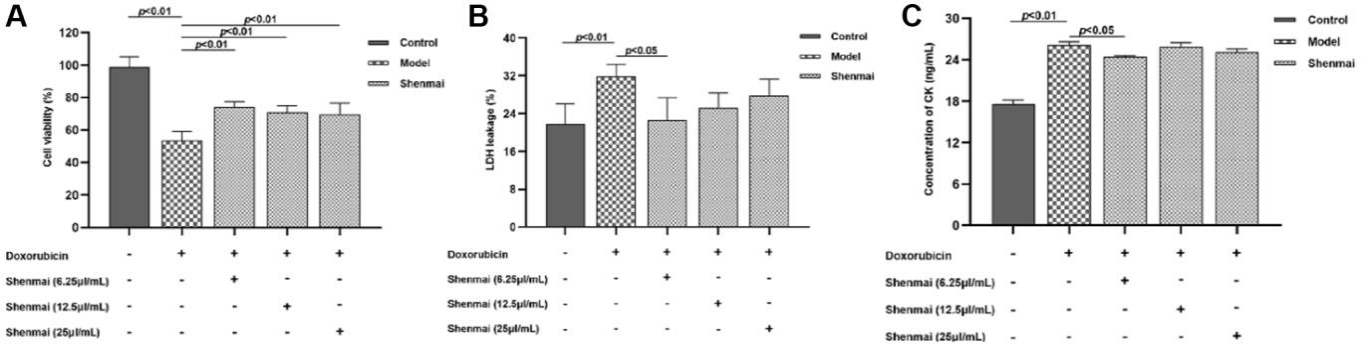
**Effects of Shenmai injection on the viability of injured H9c2 cells at different concentrations.** (**A**) The effect of Shenmai Injection on the viability of H9c2 cells; (**B**) The effect of Shenmai Injection on the LDH level of H9c2 cells; (**C**) The effect of Shenmai Injection on CK level of H9c2 cells.

CCK-8 assay was used to detect the effects of Shenmai injection at different concentrations (6.25, 12.5 and 25 μL/mL) on the viability of injured H9c2 cells. The results manifested that compared with the control group, the viability of H9C2 cells was evidently decreased in the model group (*P* < 0.01). Compared with the model group, the cell viability was significantly elevated in the Shenmai group, when the concentration of Shenmai injection was 6.25~25 μL/mL (*P* < 0.01). We further observed the effect of Shenmai injection on the degree of injury on H9c2 cells. The results turned out that compared with the control group, the levels of LDH and CK were evidently increased in the model group (*P* < 0.01). Compared with the model group, when the concentration of Shenmai injection was 6.25 μL/mL, the levels of LDH and CK were evidently decreased in the Shenmai group (*P* < 0.05), and as the concentration of Shenmai injection elevated, the levels of LDH and CK showed an upward trend (*P* > 0.05) (Figure 3A–3C). According to the above results, 6.25 μL/mL Shenmai injection was optimized for subsequent experiments.

### Effects of Shenmai injection on the viability and injury condition of H9c2 cells

**Figure 4:**
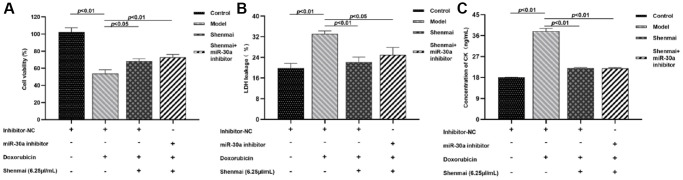
**Effects of Shenmai injection on viability of H9c2 cells.** (**A**) Effects of Shenmai Injection on the viability of H9c2 cells; (**B**) Effect of Shenmai Injection on LDH level of H9c2 cells; (**C**) Effect of Shenmai Injection on CK level of H9c2 cells.

CCK-8 assay results turned out that compared with the control group, the viability of H9c2 cells was evidently decreased in the model group (*P* < 0.01); compared with the model group, the viability elevated in the Shenmai group and Shenmai +miR-30a inhibitor group (*P* < 0.05 or *P* < 0.01). We further investigated the effect of Shenmai injection on the injury condition of H9c2 cells. The results manifested that compared with the control group, the level of LDH and CK were evidently elevated in the model group (*P* < 0.01). Compared with the model group, the level of LDH and CK declined in the Shenmai group and Shenmai +miR-30a inhibitor group (*P* < 0.01 or *P* < 0.05), indicating that Shenmai injection exerted great cardioprotective effects in doxorubicin-induced cardiotoxicity (Figure 4A–4C).

### Effects of Shenmai injection on the expression of miR-30a mRNA of H9c2 cells

**Figure 5:**
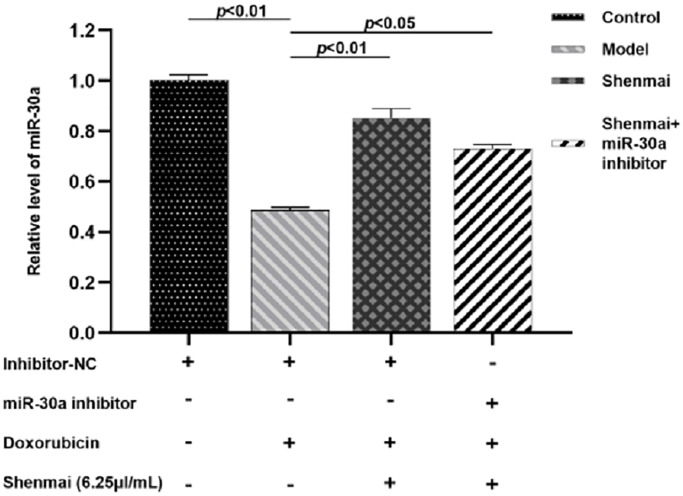
Effect of Shenmai injection on the expression of miR-30a in H9c2 cells.

Compared with the control group, the expression of miR-30a was evidently decreased in the model group (*P* < 0.01). Compared with the model group, the expression of miR-30a was elevated in the Shenmai group and Shenmai +miR-30a inhibitor group (*P* < 0.01 or *P* < 0.05), indicating that Shenmai injection could promote the expression of miR-30a in H9c2 cells (Figure 5).

### Effect of Shenmai injection on autophagy flow of H9c2 cells

**Figure 6:**
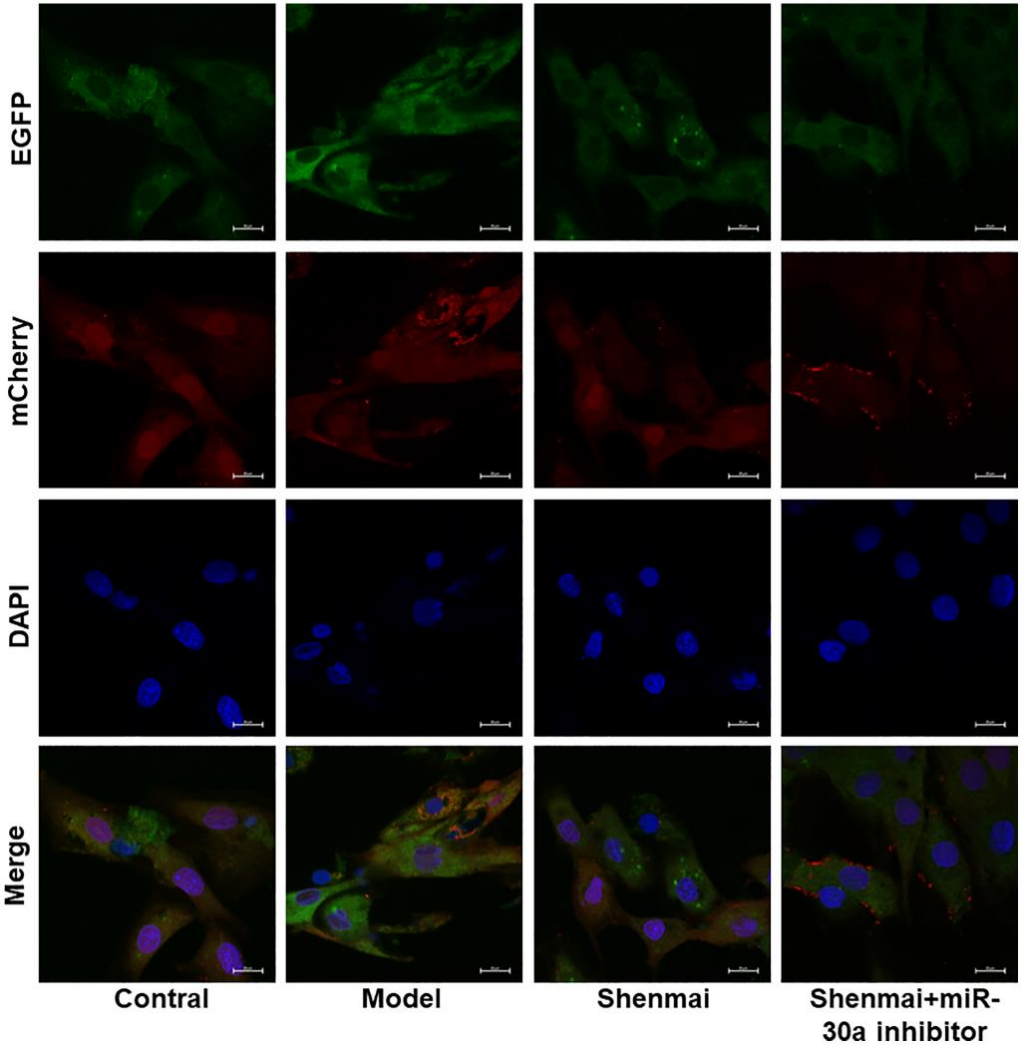
Effect of Shenmai injection on autophagy flow of H9c2 cells.

Confocal microscopy was used to observe the aggregation of mCherry-LC3B and mCherry-EGFP-LC3B in H9c2 cells. The results manifested that the red fluorescence and yellow fluorescence were not overt in the control group. Compared with the control group, there were more red fluorescence and yellow fluorescence in the model group, and the percentage of red fluorescence was relatively boosted, suggesting that there was smooth autophagy flow of H9c2 cells in the model group. Compared with the model group, the red fluorescence and yellow fluorescence reduced and the percentage of red fluorescence decreased in the Shenmai group and Shenmai+miR-30a inhibitor group, indicating that autophagy flow was inhibited and that Shenmai injection might inhibit autophagy by regulating the expression of miR-30a (Figure 6).

### Effects of Shenmai injection on the expression of autophagy-related proteins of H9c2 cells

**Figure 7:**
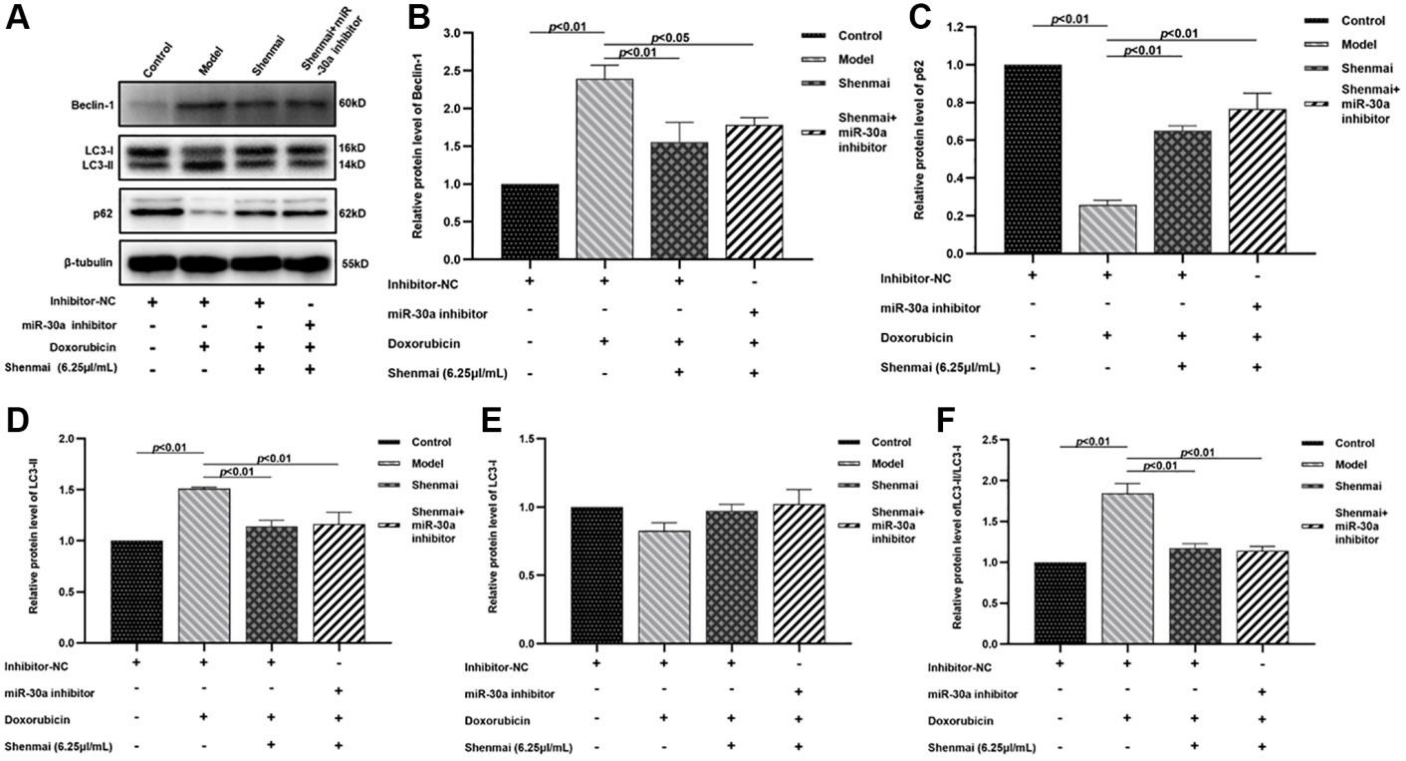
**Effects of Shenmai injection on the expression of autophagy-related proteins in H9c2 cells.** (**A**) Western Blot; (**B**) The expression of Beclin-1 protein; (**C**) The expression of p62 protein; (**D**) The expression of LC3-II protein; (**E**) The expression of LC3-I protein; (**F**) The expression of LC3-II/LC3-I protein.

Data from western blot indicated that doxorubicin contributed to the elevated autophagy, as evidenced by the decreased p62 protein level, and the increase of Beclin-1, LC3-II, and LC3-II/LC3-I levels in the model group (*P* < 0.01). Compared with the model group, the Beclin-1, LC3-II, and LC3-II/LC3-I levels were decreased (*P* < 0.01 or *P* < 0.05) accompanied by elevated p62 protein level (*P* < 0.01) in Shenmai group and Shenmai+miR-30a inhibitor group, which indicated that massive apoptosis in H9C2 cells was notably reversed by Shenmai injection and partially reversed effect on miR-30a inhibitors (Figure 7A–7F). The results indicated that Shenmai injection might inhibit autophagy by regulating the expression miR-30a.

### Effect of Shenmai injection on apoptosis of H9c2 cells

**Figure 8:**
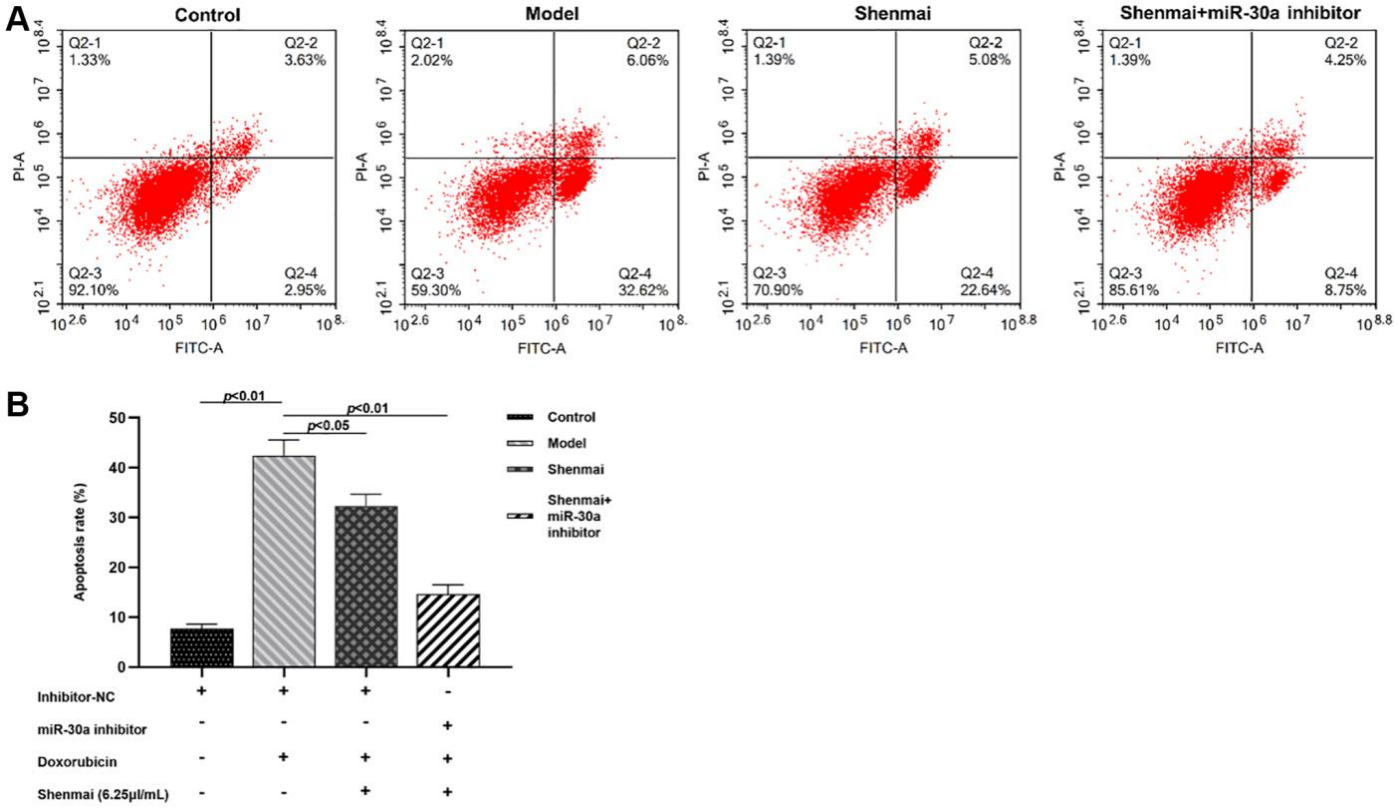
**Effect of Shenmai injection on apoptosis of H9c2 cells.** (**A**) Annexin V/PI double staining; (**B**) Effect of Shenmai injection on apoptosis rate of H9c2 cells.

Annexin V/PI double staining manifested that compared with the control group, the apoptosis rate of H9c2 cells evidently increased in the model group (*P* < 0.01). Compared with the model group, the apoptosis rate of H9c2 cells decreased in Shenmai group and Shenmai+miR-30a inhibitor group (*P* < 0.05 or *P* < 0.01), indicating that Shenmai injection might inhibit apoptosis by regulating miR-30a (Figure 8A, 8B).

### Effect of Shenmai injection on the expression of apoptosis-related proteins of H9c2 cells

**Figure 9:**
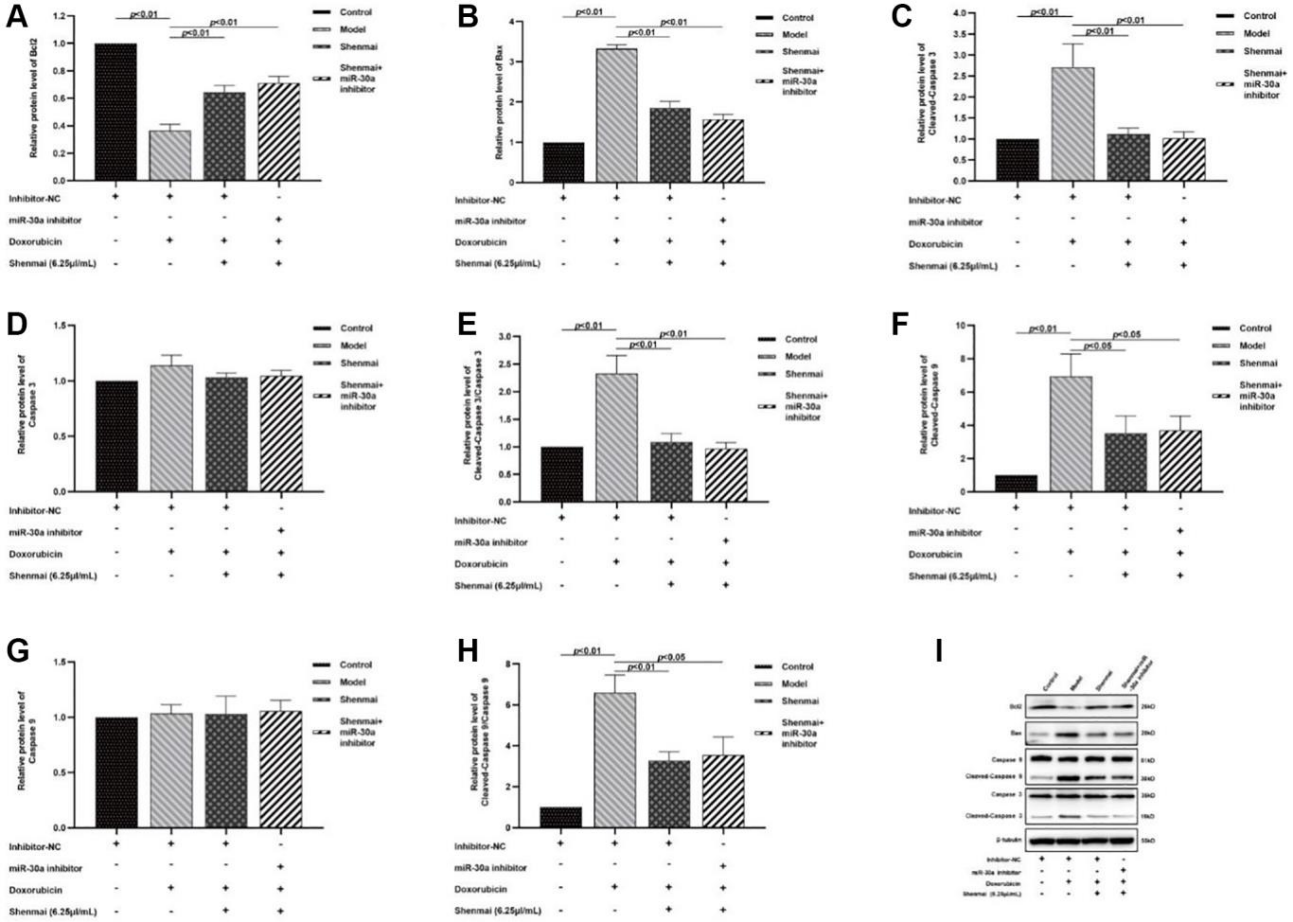
**Effect of Shenmai injection on the expression of apoptosis-related proteins in H9c2 cells.** (**A**) The expression of Bcl2 protein; (**B**) The expression of Bax protein; (**C**) The expression of Cleaved Caspase-3 protein; (**D**) The expression of Caspase-3 protein; (**E**) The expression of Cleaved Caspase-3/Caspase-3 protein; (**F**) The expression of Cleaved Caspase-9 protein; (**G**) The expression of Caspase-9 protein; (**H**) The expression of Cleaved Caspase-9/Caspase-9 protein; (**I**) Western Blot.

Data from western blot indicated that doxorubicin contributed to the elevated cardiomyocyte apoptosis, as evidenced by the down-regulation of anti-apoptotic protein Bcl2 (*P* < 0.01), and the up-regulated expression of pro-apoptotic protein Bax, Cleaved-Caspase-9, Cleaved-Caspase-9/Caspase-9, Cleaved-Caspase-3, Cleaved-Caspase-3/Caspase-3 (*P* < 0.01) in the model group. Compared with the model group, the expression of Bcl2 was up-regulated (*P* < 0.01) accompanied by the down-regulated expression of Bax, Cleaved-Caspase-9, Cleaved-Caspase-9/Caspase-9, Cleaved-Caspase-3, Cleaved-Caspase-3/Caspase-3 were in Shenmai group and Shenmai+miR-30a (*P* < 0.01 or *P* < 0.05), indicating that Shenmai injection could inhibit apoptosis of H9c2 cells and partially reverse the effect of miR-30a inhibitors. (Figure 9A–9I) The results manifested that the cardioprotective effects of Shenmai injection were mediated by regulating the miR-30a.

## DISCUSSION

Anthracyclines, the cornerstone of chemotherapy regimens, are wildly prescribed to the treatment for cancer, such as breast cancer, soft tissue sarcoma, lymphoma, leukemia and the like [17]. Whereas, irreversible cardiotoxicity induced by anthracyclines may contribute to the occurrence and development of arrhythmia, pericardial effusion, myocardial ischemia, and heart failure thanks to their property of myocardial tropism and cardiotoxicity, which reduces the survival rate and limits the clinical application of anthracyclines [18]. Study has shown that the incidence of cardiotoxicity was 26% when the cumulative dose of anthracyclines was 550 mg/m^2^; when the cumulative dose was 700 mg/m^2^, the incidence of cardiotoxicity reached up to 48% [19]. Therefore, preventive and therapeutic measures for cardiotoxicity of anthracyclines need to be developed. Over the years, an increasing number of studies focused on cardiac protectors to fight against anthracycline-induced cardiotoxicity. Among them, dexrazoan, the first cardioprotective agent approved by FDA to prevent patients from cardiotoxicity of anthracyclines, has been proven to alleviate cardiotoxicity but not the antineoplastic activity [20]. Unfortunately, there is short of conclusive evidence on the safety of dexrazoan. Research has found that dexrazoan might aggravate myelopathic depression caused by chemotherapy and increase the risk of secondary malignant tumors [21]. In addition, the protective effects of β-blockers (e.g., carvedilol and nebivolol), ACEI (e.g., enalapril), ACE (e.g., telmisartan), and N-acetylcysteine in the treatment of cardiotoxicity caused by anthracyclines remain unknown [22–25]. There still is the blank of safe and effective prevention as well as treatment for anthracyclines-induced cardiotoxicity waiting to be filled in. Traditional Chinese Medicine has shown great superiority and potential to prevent cardiotoxicity in a manner of multiple pathways, target points and elements [26]. Shenmai injection, derived from classical prescriptions, not only reduced the myocardial damage caused by doxorubicin, but also increased the antineoplastic activity, which displayed potential in the treatment of doxorubicin-induced cardiotoxicity [27]. A study involving 12 systematic reviews and a total of 28036 patients manifested that SMI reduced anthracycline-induced premature beats, atrioventricular block and other ECG abnormalities [28]. In our study, doxorubicin was used to construct injured myocardium model *in vitro*, with different concentrations of SMI (6.25, 12.5 and 25 μL/mL). The results manifested that when the H9c2 cells treated with 6.25 μL/mL SMI, the level of LDH and CK of cardiomyocytes were significantly decreased (*P* < 0.05), and with the elevated concentration of Shenmai injection, the level of LDH and CK showed an upward trend (*P* > 0.05).

Previous studies indicated that doxorubicin-induced cardiotoxicity involved in autophagy, apoptosis, oxidative stress, inflammation, mitochondrial damage, calcium homeostasis disturbance, ferroptosis and other biological processes [29]. Among them, autophagy-apoptosis is the core mechanism of doxorubicin-induced myocardial damage [30]. It was found that doxorubicin over-activated the autophagy in cardiomyocytes, caused autophagic cell death, and mediated myocardial remodeling. Doxorubicin was applied to induce myocardial damage, and the results manifested that the level of Beclin-1, a key regulator of autophagy, was significantly elevated, and LC3-II/LC3-I ratio was increased accompanied by decreased cell viability [31]. Meanwhile, doxorubicin-induced autophagy is closely related to apoptosis. Activation of Atg5 and Atg12, pivotal autophagy factors, can inhibit the expression of Bcl-2 protein, and then promote the release of cytochrome C and initiate the apoptosis process by activating protein Caspase-9 [32]. In doxorubicin-induced myocardial damage model from primary culture, autophagy flux and apoptosis rate upregulated, the expression of LC3-II, Beclin-1, PARP and Cleaved-Caspase-3 proteins increased, and the expression of p62 and Bcl2 decreased. The study further silenced the expression of Beclin-1 gene to inhibit autophagy of cardiomyocytes, and the results turned out that the survival rate of cardiomyocytes elevated, suggesting that inhibition of autophagy could ameliorate the damage of doxorubicin on cardiomyocytes [33]. In our study, mRFP-GFP-LC3 fluorescence and Annexin V/PI staining were used to observe the autophagy flow and apoptosis in H9c2 cells, and to explore the mechanism of SMI in alleviating the cardiotoxicity. The results suggested that Shenmai injection could improve doxorubicin-induced cardiotoxicity by inhibiting autophagy and apoptosis.

There is a complex interaction between autophagy and apoptosis, both mediating programmed cell death, whereas the underlying mechanism remains unclear [34]. microRNA (miRNA), the post-transcriptional regulator of gene expression, bridges autophagy and apoptosis [35]. Studies have found that miRNA could regulate autophagy and apoptosis by directly targeting upstream regulatory molecules of them [36, 37]. miRNA is involved in a variety of cardiovascular diseases such as myocardial hypertrophy and ischemia-reperfusion through regulating autophagy, apoptosis, oxidative stress and inflammation, among which miR-30a plays a pivotal role in the intersection of autophagy and apoptosis [38, 39]. In doxorubicin-induced myocardial injury rat models, the expression of miR-30a reduced, the apoptosis rate elevated, and the expression of LC3-II and Beclin-1 increased. Besides, overexpression of miR-30a improved ventricular remodeling and left ventricular diastolic function in rats [40]. In addition, doxorubicin-induced injury model *in vitro*, miR-30a expression was inhibited accompanied by declined cell viability, and the expression of p-Akt and LC3-II/I ratio were up-regulated [41]. Our study aimed at exploring the mechanism of Shenmai injection on ameliorating the cardiotoxicity of anthracyclines through regulating autophagy and apoptosis via inhibiting miR-30a expression. The results manifested that compared with model group, the expression of miR-30a and cell viability were evidently elevated; the level of LDH and CK were evidently decreased; the expression of autophagy-related proteins Beclin-1, LC3-II, LC3-II/LC3-I were up-regulated (*P* < 0.01 or *P* < 0.05); the expression of p62 was boosted (*P* < 0.01); the expression of anti-apoptotic protein Bcl2 was up-regulated (*P* < 0.01); and the expression of pro-apoptotic protein Bax, Cleaved-Caspase-9, Cleaved-Caspase-9/Caspase-9, Cleaved-Caspase-3, Cleaved-Caspase-3/Caspase-3 were down-regulated (*P* < 0.01 or *P* < 0.05), indicating that Shenmai injection exerted cardioprotective effects by interfering with autophagy and apoptosis via miR-30.

## CONCLUSION

Shenmai injection inhibited autophagy and apoptosis via miR-30a, thereby alleviating doxorubicin-induced myocardial injury.
